# Computer-Based Stratified Primary Care for Musculoskeletal Consultations Compared With Usual Care: Study Protocol for the STarT MSK Cluster Randomized Controlled Trial

**DOI:** 10.2196/17939

**Published:** 2020-07-05

**Authors:** Jonathan Hill, Stefannie Garvin, Ying Chen, Vincent Cooper, Simon Wathall, Bernadette Bartlam, Benjamin Saunders, Martyn Lewis, Joanne Protheroe, Adrian Chudyk, Hollie Birkinshaw, Kate M Dunn, Sue Jowett, Raymond Oppong, Elaine Hay, Danielle van der Windt, Christian Mallen, Nadine E Foster

**Affiliations:** 1 Institute for Primary, Community and Social Care Keele University Stoke-on-Trent United Kingdom; 2 Keele Clinical Trials Unit Institute for Primary, Community and Social Care Keele University Stoke-on-Trent United Kingdom; 3 Nanyang Technological University Singapore Singapore; 4 Health Economics Unit Institute of Applied Health Research University of Birmingham Birmingham United Kingdom

**Keywords:** musculoskeletal, primary health care, stratified care, randomized controlled trial, therapeutics, economics, outcome and process assessment, health care, prognosis, qualitative research, back pain, osteoarthritis

## Abstract

**Background:**

Musculoskeletal (MSK) pain is a major cause of pain and disability. We previously developed a prognostic tool (Start Back Tool) with demonstrated effectiveness in guiding primary care low back pain management by supporting decision making using matched treatments. A logical next step is to determine whether prognostic stratified care has benefits for a broader range of common MSK pain presentations.

**Objective:**

This study seeks to determine, in patients with 1 of the 5 most common MSK presentations (back, neck, knee, shoulder, and multisite pain), whether stratified care involving the use of the Keele Start MSK Tool to allocate individuals into low-, medium-, and high-risk subgroups, and matching these subgroups to recommended matched clinical management options, is clinical and cost-effective compared with usual nonstratified primary care.

**Methods:**

This is a pragmatic, two-arm parallel (stratified vs nonstratified care), cluster randomized controlled trial, with a health economic analysis and mixed methods process evaluation. The setting is UK primary care, involving 24 average-sized general practices randomized (stratified by practice size) in a 1:1 ratio (12 per arm) with blinding of trial statistician and outcome data collectors. Randomization units are general practices, and units of observation are adult MSK consulters without indicators of serious pathologies, urgent medical needs, or vulnerabilities. Potential participant records are tagged and individuals invited using a general practitioner (GP) point-of-consultation electronic medical record (EMR) template. The intervention is supported by an EMR template (computer-based) housing the Keele Start MSK Tool (to stratify into prognostic subgroups) and the recommended matched treatment options. The primary outcome using intention-to-treat analysis is pain intensity, measured monthly over 6 months. Secondary outcomes include physical function and quality of life, and an anonymized EMR audit to capture clinician decision making. The economic evaluation is focused on the estimation of incremental quality-adjusted life years and MSK pain–related health care costs. The process evaluation is exploring a range of potential factors influencing the intervention and understanding how it is perceived by patients and clinicians, with quantitative analyses focusing on a priori hypothesized intervention targets and qualitative approaches using focus groups and interviews. The target sample size is 1200 patients from 24 general practices, with >5000 MSK consultations available for anonymized medical record data comparisons.

**Results:**

Trial recruitment commenced on May 18, 2018, and ended on July 15, 2019, after a 14-month recruitment period in 24 GP practices. Follow-up and interview data collection was completed in February 2020.

**Conclusions:**

This trial is the first attempt, as far as we know, at testing a prognostic stratified care approach for primary care patients with MSK pain. The results of this trial should be available by the summer of 2020.

**Trial Registration:**

ISRCTN Registry ISRCTN15366334; http://www.isrctn.com/ISRCTN15366334.

**International Registered Report Identifier (IRRID):**

DERR1-10.2196/17939

## Introduction

### Background

Musculoskeletal (MSK) pain from common conditions such as back pain and osteoarthritis is a major cause of pain and disability. Estimates from the most recent global burden of disease study suggest that it is the leading cause of disability-adjusted life years (DALYs) in Western Europe and Australia [1]. Overall, it accounts for 6.8% of global DALYs, comparable with cancer (7.8%), ischemic heart disease (5.2%), and mental health disorders (7.4%). This burden is reflected in health care use, particularly in UK primary care where MSK pain accounts for around one-fifth of all consultations [2-4]. It also accounts for 8.8 million physiotherapy consultations and over 3.5 million calls annually to emergency services [5]. Usually, general practitioner (GP) care for MSK pain involves a long-term management approach carried out during short 10-min face-to-face consultations during which patients are assessed and treated with advice, education and reassurance, analgesic medication, referral for investigation(s), referral to other services offering conservative treatments such as physiotherapist-led exercise, or referral to secondary care medical specialists such as orthopedic consultants and rheumatologists. For many patients, primary care clinicians should reassure them that their MSK pain is not associated with serious underlying pathology, that the prognosis is usually good, and that further tests are not indicated, combined with advice and support to help them stay active [6]. However, evidence suggests substantial variability in clinical practice, with treatment often not in line with best practice recommendations in guidelines, particularly with respect to opioid medication and x-ray investigation [7].

Due to the high prevalence of these common symptoms, MSK pain has overtaken mental health issues such as stress as the number one reason why people take time off work in Europe and the United States [1]. The early identification and improved management of those at risk of severe disabling MSK pain in primary care, where the majority of these patients are managed, is therefore a high priority [8]. Patients with different MSK pain presentations (eg, back, neck, knee, shoulder, or multisite pain) share common prognostic factors [9]. Co-occurrence of MSK pain located in more than one body region is common [10], with the risk of a poor outcome increasing for those with multisite pain [11]. For example, the Chronic Pain Risk Score [12] has been shown to have predictive validity among patients with MSK pain in different body regions [13-15]. However, previous prognostic questionnaires such as the Chronic Pain Risk Score and the Orebro-MSK Pain Screening Questionnaire [16] were not designed to guide primary care management, and their use in primary care clinical practice is uncommon.

Consequently, we previously developed a prognostic tool (Start Back Tool) specifically for use in primary care to guide the management of patients with low back pain [17]. Prognostic stratified care models involve matching treatments to the patient’s prognostic profile to support clinical decision making in an effort to maximize treatment benefits, reduce harm, and increase health care efficiency [18]. The Start Back Tool consists of 9 questions summed into an index score. It utilizes cutoff points to identify 3 prognostic subgroups (patients at low, medium, or high risk of persistent disabling pain). In 2 previous UK studies, stratified care for back pain, based on matching treatment to prognosis, led to superior clinical and economic outcomes compared with best current practice and usual primary care [19,20]. The evidence suggested that patients at low risk received fewer investigations and referral to secondary care, and in contrast, patients at medium or high risk were matched to treatments that could better meet their needs, leading to improved outcomes.

### Rationale

A logical next step is to determine whether a similar model of prognostic stratified care might also have benefits for primary care patients with a much broader range of MSK pain presentations. The 5 most common MSK pain presentations in UK primary care are low back pain, knee pain, shoulder pain, neck pain, and multisite pain [2]. In a research program with 4 work packages (the Start MSK program), our team first developed and validated a new 10-item prognostic tool, the Keele Start MSK Tool, to stratify patients with the 5 most common MSK pain presentations into subgroups (those at low, medium, and high risk of persistent pain and disability) [21]. Second, we agreed on evidence-based recommended matched treatment options for patients in each subgroup following a systematic review [22] and expert consensus process [23]. Third, we conducted an external feasibility and pilot randomized trial with 524 patients from 8 general practices (4 intervention and 4 control) [21,24]. The pilot trial confirmed the acceptability of using a stratified care approach in primary care consultations and also helped to refine our recruitment, retention, and sample size estimates, ahead of the main trial. The findings informed the final wording of the self-report version of the Start MSK Tool, led to a clinician-completed version of the tool, and allowed us to simplify the recommended matched treatment options. All changes made to the main trial protocol following the pilot trial were discussed, shared, and agreed with the trial funder the National Institute for Health Research (NIHR), the Trial Steering Committee (TSC), and the Data Monitoring Committee (DMC).

### Aims and Objectives

#### Primary Objective

The primary objective of the Start MSK main trial is to determine, in patients presenting with 1 of the 5 most common MSK pain presentations in UK primary care, whether stratified care involving the use of the Keele Start MSK Tool to allocate individuals into low-, medium-, and high-risk subgroups, and matching these subgroups to recommended matched clinical management options, is more clinically and cost effective compared with usual nonstratified primary care. The primary clinical outcome is average pain intensity over the past 2 weeks measured each month for 6 months.

#### Secondary Objectives

The secondary objectives of the trial were as follows:

1. Examining differences in secondary clinical outcomes, clinical decision making and behaviors, and health economic outcomes at the 6-month follow-up:

- Patient outcomes include physical function, confidence in managing their pain (pain self-efficacy), psychological distress, fear-avoidance beliefs, patient-perceived reassurance from their clinician, pain interference with sleep, hobbies/leisure activities, pain interference with work and daily routine, health-related quality of life, and patient satisfaction with care received.
- Clinical decisions and behaviors of interest include identifying whether stratified care changes the primary care management of MSK patients. We anticipate that primary care clinical management will become more consistent for patients within each risk group and be more in line with stratified care, where patients at low risk of persistent disabling pain are less likely to be referred for additional health care, whereas patients at medium or high risk are more likely to be referred for additional health care in ways that match the recommended management options. Using the practices’ medical record data, we will examine differences between the trial arms in clinical decision making and behaviors.
- Health economic evaluation will determine the cost-utility of stratified care in comparison with usual, nonstratified care. A cost-consequence analysis will initially be reported, with a subsequent cost-utility analysis from a health care perspective to determine cost per quality-adjusted life years (QALYs) gained, calculated using EuroQol-5D-5L (EQ-5D-5L) responses from the initial and 6-month questionnaires. A broader costing perspective will be considered in a sensitivity analysis, taking into account National Health Service (NHS)/Personal Social Services (PSS) costs and productivity costs associated with time off work. The outcome of interest for the economic analysis will be QALYs. Additional exploratory analyses will consider the cost-effectiveness of stratified care compared with usual nonstratified care for patients at low, medium, and high risk of persistent disabling pain.

2. Undertaking a process evaluation to explore how stratified care, as a complex intervention, interacts with existing patterns of service organization, professional practice, and professional-patient interaction. The evaluation will use mixed quantitative (eg, a mediation analysis) and qualitative methods, integrating data both at the collection and analysis stages, to generate more detailed and comprehensive findings.

## Methods

### Study Design and Setting

**Figure 1:**
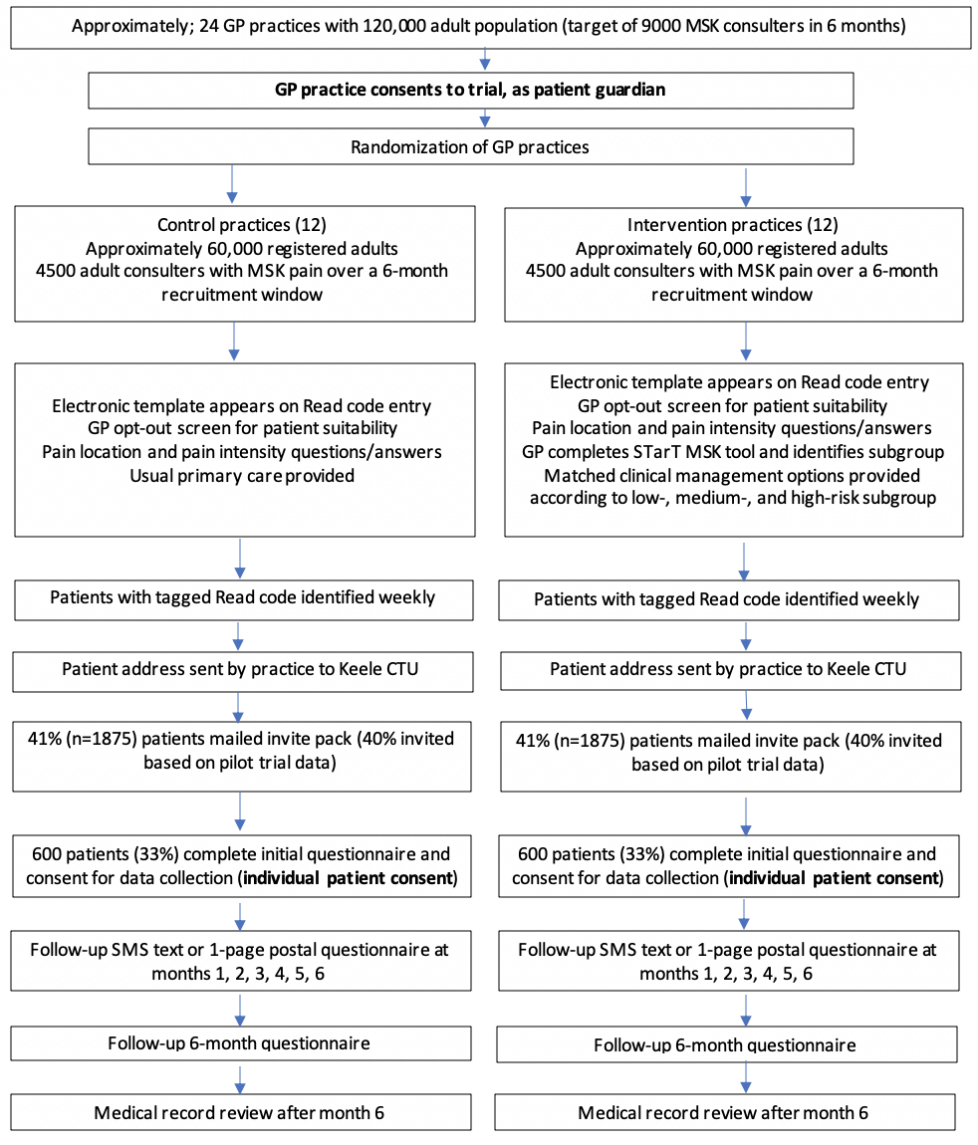
Flowchart of patient recruitment. CTU: clinical trials unit; GP: general practice; MSK: musculoskeletal.

The Start MSK trial is a pragmatic, two-arm, parallel, cluster randomized controlled trial (RCT), with a linked health economic analysis and mixed methods process evaluation. The setting is UK primary care, and the trial will include approximately 24 average-sized general practices with a total registered adult population of approximately 120,000. General practices will be randomized to either the stratified care intervention (12 practices) or the usual, nonstratified care (12 practices). The units of randomization are the general practices, and the units of observation are adults consulting for MSK pain with 1 of the 5 most common MSK pain presentations (Figure 1).

The intervention in both arms of the trial will include an embedded template within the general practice computer system, which will *pop-up* during the first relevant Read-coded MSK pain consultation within the specified study period (termed the *MSK consultation*; this may be the first consultation or a repeat consultation for MSK pain). However, the content of the template will differ between the 2 arms of the trial. In the control arm, it includes 3 questions: (1) the eligibility of the patient to be invited to participate, (2) the location/site of MSK pain for which the patient is consulting, and (3) the average MSK pain intensity in the past 2 weeks (primary outcome). In the intervention arm, in addition to these 3 questions, the template also includes the Keele Start MSK Tool and recommended matched treatment options [21].

A cluster RCT rather than an individual patient RCT was chosen for both scientific and practical reasons. Stratified care is a new way of working, and the tool, training, and support are delivered at the general practice level (eg, the computer template once installed will *pop-up* on all computers in practice). Primary care clinicians would likely find it difficult to behave differently toward individuals randomized to control and intervention arms, and therefore, the probability of contamination between the 2 arms would be high using an individual patient randomized trial design. This trial can be thought of as a professional-cluster intervention type [25], in that the stratified care intervention involves changing the professional’s behavior during the consultation, in this case, using a prognostic tool and matching patients to clinical management options. Although the patient can opt out of data collection, the intervention is still likely to have an effect on them as it involves introducing specific questions and recommendations about matched treatment options into the consultation.

### Minimizing Systematic Bias

The risk of selection bias, specifically of recruitment and participation bias, is a known concern in cluster RCTs [26]. A number of steps have been taken to minimize this, which were tested in the pilot RCT, where we observed no evidence of selection bias:

- The initial part of the computer template to help identify eligible patients is automated based on diagnostic codes entered during the consultation and operates in the same way in both arms of the trial.
- If the clinician deems a patient to be ineligible, they are asked to give a reason for this exclusion so that these reasons can be compared across intervention and control arms. This process is monitored during trial recruitment, with monthly feedback provided to participating practices showing the frequency of template noncompletion, and the proportions of different reasons for ineligibility.
- Patients in both arms of the trial will receive identical study invitation packs comprising the same patient information leaflet (PIL; which does not mention stratified care, only that the study seeks to better understand how common aches and pains affect patients and how primary care can be improved), invitation letter, questionnaire, and consent form for data collection, minimizing the risk of patients in intervention or control arms being more or less likely to participate (participation bias).

### General Practice Recruitment and Consent

It is anticipated that an estimated eligible target population of approximately 9000 patients will be identified within a 6-month recruitment window from approximately 24 average-sized general practices (approximately 120,000 registered adults). Practices will include those that range in size (based on patient list size and number of GPs) and a range of settings (urban, semi-urban, and rural). The practice eligibility criteria includes those that use the Egton Medical Information Systems (EMIS) web clinical system (most commonly used electronic medical record [EMR] system in the United Kingdom), those proficient at using Read codes (diagnostic codes) during MSK consultations evidenced through an audit of their recent Read coding behavior, willingness to undergo the training and support sessions needed to become familiar with the stratified care intervention, willingness to participate in anonymized aggregated medical record audits of MSK consultations during the trial recruitment period, and willingness to engage with the process evaluation.

The balance between scientific considerations and the need for consent is a known issue for cluster RCTs [25,26]. Informed consent for practices to participate is formalized through written agreements led by the senior GP partner in each practice acting as a *guardian* for patients in their care, following agreement with their team to provide either usual care or stratified care for the period of the trial (dependent on random allocation). It is anticipated that practices will actively recruit patients for approximately 6 months, with practice recruitment periods staggered over a 12-month period. Reimbursement for the practice time to recruit and participate in the training is provided.

### Individual Patient Participants

Potential individual patient participants will consult at a participating practice with 1 of the 5 most common MSK pain presentations (back, neck, knee, shoulder, or multisite pain) as determined by the clinician at the point of consultation.

Patient inclusion criteria were aged 18 years and over, registered at the practice during the recruitment period, with a recorded relevant MSK pain Read code entered into the computer system (this may be the first or a repeat consultation), a completed study template, consent to study data collection, consent for research team to have access to their medical record data, and completion of the initial postal questionnaire within 4 weeks of the first mailing.

Patient exclusion criteria were those with indications of serious red flag pathology (eg, recent trauma with significant injury; acute, red, hot swollen joint; suspected fracture; joint infection; cancer; and inflammatory arthropathy such as rheumatoid arthritis, spondyloarthropathy, polymyalgia rheumatica, and crystal disease [gout]), those with urgent medical care needs (eg, cauda equina syndrome), vulnerable patients (including any patients on the severe and enduring mental health register, those who have a diagnosis of dementia, those with a recent diagnosis of a terminal illness, those who have experienced recent trauma or bereavement, or those nearing the end of their life), and those who are unable to communicate in English (both in reading and speaking).

### Patient Recruitment for Outcome Data Collection

**Figure 2:**
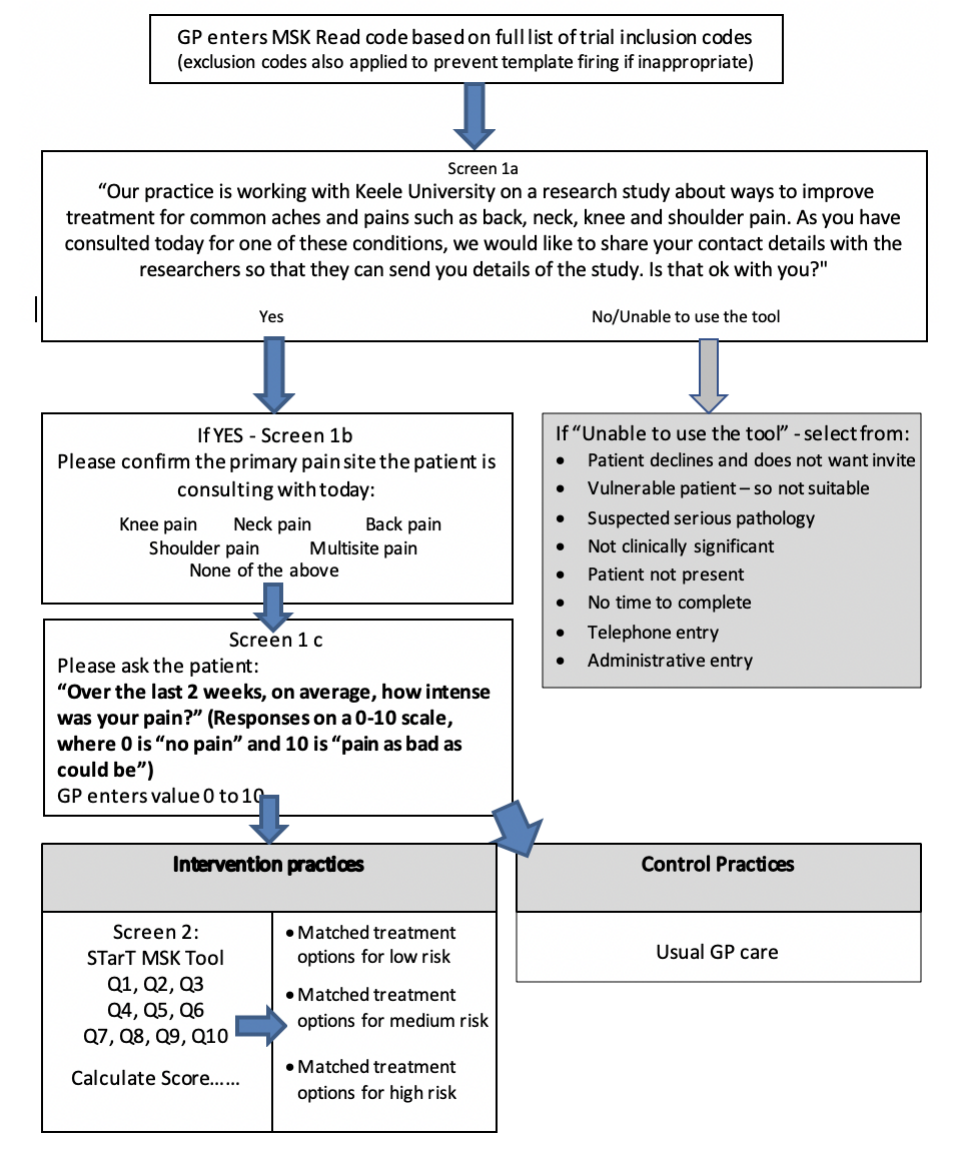
Details of the trial recruitment template. GP: general practice; MSK: musculoskeletal; Q: question.

As described above, an electronic computer template designed to automatically fire to help identify patients will be installed on participating practice computer systems, when 1 of approximately 200 different MSK pain–related Read codes (symptom or diagnostic codes) is entered, as defined by Jordan et al [2] and informed by our pilot RCT (Figure 2). Clinicians will be trained to use this system, but it is already standard practice in NHS primary care since 1985 for clinicians to use this standard vocabulary to record patient findings and procedures in health and social care Information Technology (IT) systems across the United Kingdom. In the consultation, when an MSK-related Read code is entered onto the EMR system, the trial-specific template will be activated. Initially, it prompts the clinician to notify eligible patients about the research study by reading the following:

Our practice is working with Keele University on a research study about ways to improve treatment for common aches and pains such as back, neck, knee and shoulder pain. As you have consulted today for one these conditions, we would like to share your contact details with the researchers so that they can send you details of the study. Is that ok with you?

Patients who do not give this consent do not have their contact details shared with the research team. Individuals who have previously asked not to be part of any research within the practice are given a Read code that prevents the template from firing in the first place. Retention of identifiable patient data is restricted to the limited period of invitation only, after which, the data of subsequent nonconsenters to the trial will be destroyed. The Keele Clinical Trials Unit (CTU) operates this activity in compliance with the provisions of the Data Protection Act (1998) and adheres to appropriate standards of governance and security as outlined by the sponsor’s (Keele University) standard operating procedures (SOPs).

Practice staff supported by the NIHR Clinical Research Network (CRN), where possible, will regularly (typically weekly) send the contact details of patients for whom the template has been successfully completed to Keele CTU for the purpose of mailing patients their invitation letter, using a secure NHS.net email to transfer data including name, address, Read code for pain site, date of consultation, and EMIS patient identification number. On a monthly basis, the participating practices provide anonymized information about the number of patients for whom the study template is activated and how many of those are not fully completed. This facilitates the provision of a monthly report of their template completion rate and, for intervention practices, additional details about their fidelity to choosing recommended matched clinical management options for patients at low, medium, and high risk.

Eligible participants (from both trial arms) will be sent identical study packs in the post containing a letter from the patient’s general practice introducing the study; a PIL, which describes the study and includes instructions on what to do if they wish to take part; an initial questionnaire, including a consent form to record consent for data collection; and a stamped addressed envelope. The following mechanisms will ensure that only eligible and appropriate patients are invited: (1) a list of relevant exclusion Read codes (eg, recent cancer diagnosis) will be used to automatically prevent the template from firing and (2) clinicians will be able to screen individual patients for their suitability at the point of consultation.

In the initial questionnaire sent to patients, participants will provide their written consent for researchers to use their data for this research. A study team member (blind to practice allocation) will support patients who telephone with questions or who need additional support to complete their postal questionnaires, monthly SMS texts, or 1-page questionnaires. The same set of MSK Read codes that trigger the automated template was successfully used as the identification method in our pilot RCT. The electronic identification method is designed to ensure that the template *pop-up* is only activated once per patient, so individuals can only be invited once to participate in this study. Eligible patients who do not respond within 2 weeks of their initial study invitation will be contacted again with another study pack. Patients who do not complete their initial questionnaire within 4 weeks of the initial mailing date will not be contacted again for follow-up data. Patients who return their initial questionnaire and consent to further data collection will be included in the study. The primary outcome (pain intensity) will be collected once a month for 6 months via SMS text or 1-page postal questionnaire (depending on participant preference).

The procedures for reminders for the SMS text monthly communications are as follows: the initial contact will be sent on the next Sunday afternoon that is closest to a calendar month following their initial questionnaire mailing date. If there is no response to this initial contact, a reminder communication will be sent on Tuesday afternoon. If again, there is no response after 48 hours, we will send the monthly 1-page postal questionnaire. On the second consecutive month, we will repeat this procedure; however, if there is no response, in addition to sending the monthly 1-page postal questionnaire, a study team member will telephone the patient to establish what the problem is, seek to resolve it, provide appropriate support, and collect the data where possible. For those receiving the monthly 1-page postal questionnaire, nonresponse after 2 weeks will lead to another 1-page postal questionnaire. Nonresponse on a second consecutive month will lead to a study team member telephoning the patient to establish what the problem is, seek to resolve it, provide appropriate support, and collect the data where possible. Participants will also receive a 6-month follow-up questionnaire to collect further outcomes. Nonresponders to the 6-month follow-up questionnaire will be sent a reminder postcard at 2 weeks and a full questionnaire 2 weeks later (ie, at 4 weeks), and for those who have not responded, a brief questionnaire will be sent after 6 weeks to collect key outcome measures. We will try to collect minimum data over the telephone from participants at 8 weeks where needed. These follow-up methods have been used successfully in previous studies [27-30], including the pilot RCT.

### Randomization and Blinding

Practices will be randomized in a ratio of 1:1 to intervention or control using stratified block randomization [31] based on practice patient list size using a Keele CTU computer-generated random sequence and concealment by ensuring that each practice has an anonymized code. The randomization sequence and stratification will be carried out by the senior trial statistician. The block randomization will follow Keele CTU’s randomization SOP, and the data sequence will be held on a secure server. Blinding for individual clinicians is not possible, but any staff involved in the collection or database entry of patients’ outcome data will be blind to allocation. Access to the allocation sequence will be restricted to those with authorization. Allocation will be shared with the study team (except for the trial statistician and data entry staff who are to remain blind) who will then arrange to inform each practice about their allocation. Data cleaning/checking through stage 1 *data-freeze* and stage 2 *data-lock* reviews will be carried out by the trial statistician, thus maintaining blinding to allocation. The TSC will also be blinded to allocation unless it becomes absolutely necessary to reveal allocation. The DMC trial statistician will be involved in the allocation assignment and, therefore, will not be blinded throughout the study. These processes follow recommendations for cluster trials [26] to reduce selection bias where randomization is before patient data collection.

### Interventions

#### Practice Recruitment Template Installation

Following confirmation that a practice is eligible and willing to take part, an initial setup meeting will be held between the practice, study research team, and a CRN member. This will take place for all practices in both arms of the trial and will be followed by training sessions where the computer template will be installed and demonstrated on the practice’s EMIS clinical system. Once the template is installed, the practice is *live*, and potentially eligible participants will be identified in consultations.

### Intervention Arm

The recommended matched clinical management options are not new but summarize available evidence-based options into those considered by expert consensus to be appropriate for patients at low, medium, and high risk of persistent pain and disability.

The Start MSK stratified care approach has 2 components: (1) prognostic tool and (2) matched options.

#### Prognostic Tool

The Keele Start MSK Tool (clinician-completed version) is freely available [32]; is used in the patient consultation [21]; and is supported by an embedded template in the practice’s computer system, dedicated training and support sessions, regular audits, peer feedback, and clinical mentoring opportunities using an evidence-based clinician support package to support clinician behavior change [33]. The prognostic tool has 10 questions from which the patient’s score and subgroup (low, medium, or high risk of persistent pain and disability) are calculated.

#### Matched Options

Appropriate matched clinical management options based on an individual’s prognosis on the Keele Start MSK Tool will be displayed to support clinical decision making. The matched clinical management options were identified by an evidence synthesis [22], followed by 3 expert consensus workshops [23], during an earlier phase of research, and then further refined following the Start MSK feasibility and pilot [21].

### Per Protocol Treatment Decision Rules

**Figure 3:**
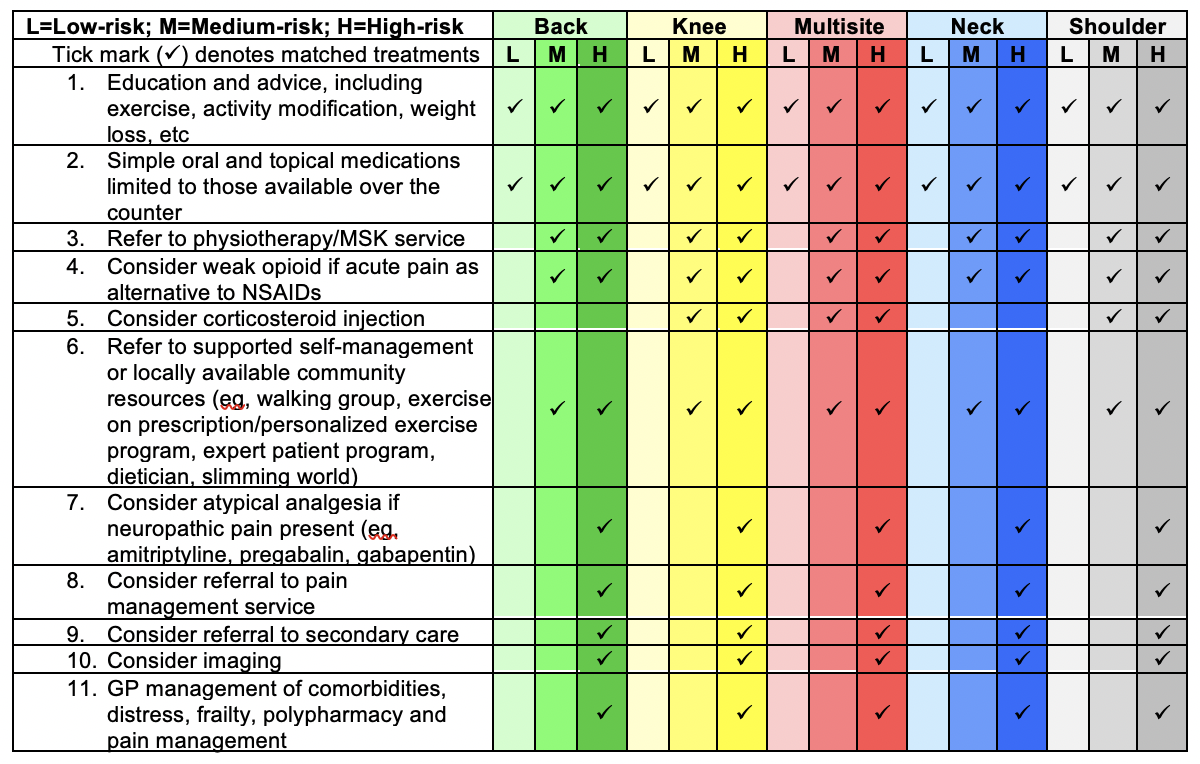
Recommended matched treatment options. GP: general practice; MSK: musculoskeletal; NSAIDS: Nonsteroidal anti-inflammatory drugs.

Patients at low risk will be considered to be treated *per protocol* if they receive only treatment options 1 or 2 (Figure 3). Patients at medium risk will be *per protocol* if they receive any of options 3-6 (although option 5 is for specific pain sites only). Patients at high risk will be *per protocol* if they receive option 3 or any options between 7 and 11.

The matched options for patients at low risk include advice and education (using printed materials where possible), over-the-counter analgesics, and avoidance of MSK investigations and referrals (where possible). Matched options for patients at medium risk, in addition to the low-risk options, include GPs being encouraged to refer patients to physiotherapy, to review their pain medication, and to consider investigations where necessary. Matched options for patients at high risk, in addition to the medium- and low-risk options, include prescription of atypical analgesia if neuropathic pain is present; referral to specialist services (eg, orthopedics, rheumatology, and pain clinics); imaging; and/or booked reviews to manage complex clinical factors such as comorbidities, polypharmacy, and frailty.

### Clinician Support for Delivering Stratified Care

The training and support sessions provided to intervention and control clinicians are designed to equip them with the knowledge and skills to complete the study recruitment template and understand the study inclusion/exclusion criteria. In addition, for intervention practices, a 2-hour intervention training session will be provided. This includes learning about previous stratified care research [19,20], the rationale for developing this new intervention, and investigating whether it will benefit patients with a broader range of MSK pain. Training will describe the aim to reduce unnecessary health care for patients at low risk, while better targeting health care resources for patients at medium or high risk. Clinicians will have a demonstration of how to use the new approach and have the opportunity to try it out and ask questions and explore how it can be integrated into routine practice. The session will also include discussion and clarification about how the approach differs from usual care and each of the recommended clinical management options. We will also invite a representative from the local MSK physiotherapy service to the training sessions and discuss how best to ensure that patient referrals to physiotherapy include a record of the index consultation and the patient’s Start MSK Tool risk group and determine the best method for physiotherapists to communicate with referring GPs if they are concerned about the patient. A feedback meeting will be held with all participating practices (intervention and control) roughly 6 weeks after starting recruitment to discuss the report of their use and completion of the study-specific IT template. For intervention practices, additional feedback on their fidelity to the recommended matched clinical management options will also be provided, comparing each clinician with their colleagues in the same practice and with other clinicians in the trial (anonymized). Monthly email feedback reports will be sent to participating practices.

Physiotherapists linked to intervention practices will also have the opportunity to attend a short training session about the trial and be required to avoid treating patients from control practices for the period of the trial to avoid contamination. However, other key features of physiotherapy care will be as similar as possible for patients irrespective of whether they come from intervention or control practices, including physiotherapy waiting times, treatment session length and number, and the clinical grade of the treating therapist. We will collect these process data from physiotherapy services using a mix of usual clinical record data and standardized case report forms for the study.

### Control Arm

In the usual care control arm, patients who consult at their general practice will be assessed and receive advice and treatment as usual (eg, advice and education; medication; referral for investigations or tests; or referral to other services such as physiotherapy, MSK interface clinics, or secondary care specialists such as orthopedics and rheumatology), without the use of formal stratification tools. To keep the control arm as close to *usual care* as possible, clinicians will be advised to follow their usual approach for responding to a patient’s pain intensity rating for the presenting MSK problem. Asking a patient the intensity of their pain and where their pain is coming from is common practice [30] and therefore should have little impact on the *usual care* provided.

### Data Collection

There will be 3 different types of data collection:

1. Individual patient data, collected from:

- The practices’ completed computer templates at the point of consultation
- Initial and 6-month postal questionnaires to participants (full and minimum data versions)
- Monthly SMS text or 1-page postal questionnaire.

2. Clinician decision making and behaviors using data collected from medical records and case report forms

3. Practice-level anonymized aggregated data of MSK Read codes and template use.

### Individual Patient Outcomes

#### General Practice Information Technology Template

The first item on the template asks the primary care clinician to confirm if their patient gives consent to have their contact details shared with the research team. If the answer is yes, then the clinician will record the location of the patient’s MSK pain. How this is answered determines which study letter and questionnaire the patient will be sent, as these are slightly different for patients with back, neck, knee, shoulder, or multisite pain. The item reads “Please confirm the primary pain site the patient is consulting with today.” Possible response options include *back pain*, neck pain, *knee pain*, *shoulder pain*, *multisite pain*, or *unable to complete template* (which leads to the exit screen). The third question on the template asks the clinician to record the patient’s MSK pain intensity by asking “How intense was your pain, on average, over the last 2 weeks?” (Responses are recorded on a scale of 0-10, where 0 is *no pain* and 10 is *worst pain ever*.)

#### Initial and 6-Month Follow-Up Questionnaires

The initial and 6-month follow-up postal questionnaires are designed to collect information on descriptive characteristics of the participants, pain-related characteristics, and primary and secondary outcome measures (see Table 1 below). Patients are informed in their study invitation that they have been contacted because they recently visited their general practice (the date of their visit will be given) for their MSK pain, which will be prepopulated in the letter (eg, knee pain, shoulder pain) using information from weekly downloaded template codes.

Participants will also be told that it is important they think about their *MSK pain* as they answer the following question: “Thinking about your (eg, neck) pain: Over the last 2 weeks, on average, how intense was your pain?” (Responses were recorded on a scale of 0-10, where 0 is *no pain* and 10 is *worst pain ever*.)

**Table 1:** A summary of patient-reported data collected.

Conceptual domain		Operational definition	Empirical measure	Number of items	Time
**Primary outcome**					
	Pain intensity	Usual pain intensity	NRS^a^ 0-10	1	GPT^b^, I^c^, 6FU^d^, MF^e^, and MDC^f^
**Secondary outcomes**					
	Risk status—Start MSK^g^ Tool	Risk of persistent disabling pain	Yes/no	9	I and 6FU
	MSK health	Impact from MSK symptoms	MSK-HQ^h^, 5-point Likert scale	14	I and 6FU
	Overall rating of change	Change since MSK pain consultation	−5 to +5 scale	1	I and 6FU
	Physical activity level	Days past week of moderate activity	1-7 days	1	I and 6FU
	Fear-avoidance beliefs	Fear of movement	Tampa Scale of Kinesiophobia-11	11	I and 6FU
	Satisfaction	Satisfaction with care	5-point Likert scale	1	I and 6FU
	Perceived reassurance from general practitioner consultation	Patient-perceived reassurance 4 subscales: information gathering, relationship building, generic reassurance, and cognitive reassurance	12 items with 7-point Likert scale	12	I
	Receipt of written education material from general practitioner	Single item to ask if patient received written information at their general practitioner visit	Yes/no/don’t remember	1	I
	Pain self-efficacy	Single item: confidence to manage pain	NRS 0-10	1	I and MF
	Psychological distress	Single item regarding level of distress	NRS 0-10	1	I and MF
**Site-specific function depending on MSK pain site**					
	Back pain function	Physical function	RMDQ^i^ – original version	24	I and 6FU
	Neck pain function	Physical function	NDI^j^	10	I and 6FU
	Shoulder pain function	Physical function	SPADI^k^	13	I and 6FU
	Knee pain function	Physical function	KOOS-PS^l^	7	I and 6FU
	Multisite pain function	Physical function	SF-12 PCS^m^	12	I and 6FU
**Quality of life measures**					
	Health-related quality of life	Utility-based quality of life	EuroQol-5D	5	I, 6FU, and MDC
**Health care costs**					
	Health care resource use	Use of primary care, other National Health Service services, and private health care	Yes/no and if yes details of resources used	3	6FU
	Performance at work	How productivity at work is affected	NRS 0-10	1	I and 6FU
	Absenteeism from work	Number of days absent from work	Yes/no and details	1	I and 6FU
**Patient descriptors**					
	Age	Age at MSK consultation	Date of birth	1	GP T
	Sex	Sex	Male/female	1	GP T
	Employment status and absence from work	Employment status at time of questionnaire	Yes/no and details	1	I and 6FU
	Socioeconomic status	The individual’s current or most recent job title	Job title: categorized as manual/nonmanual	2	I
	MSK pain location	Site of MSK pain complaint	Choice of anatomical region	1	GP T
	Episode duration	Time since last whole month pain free	Episode duration	1	I
	Health literacy screen	Health literacy	Single question: Likert scale	1	I
	Comorbidities	Other diagnosed comorbidities to select from a list	Yes	1	I
	Support needed	Who has completed the questionnaire	Patient/carer/staff/other	1	I
	Living arrangements	Lives alone	Yes/no	1	I
	Previous episodes	Number of previous pain episodes	Number	1	I

^a^NRS: numerical rating scale.

^b^GP T: GP template.

^c^I: initial questionnaire.

^d^6FU: 6-month follow-up.

^e^MF: monthly follow-up.

^f^MDC: minimal data collection.

^g^MSK: musculoskeletal.

^h^MSK HQ: Musculoskeletal Health Questionnaire.

^i^RMDQ: Roland-Morris Disability Questionnaire.

^j^NDI: Neck Disability Index.

^k^SPADI: Shoulder Pain and Disability Index.

^l^KOOS-PS: Knee Injury and Osteoarthritis Outcome Score-Physical Function Shortform.

^m^SF-12 PCS: Short-Form 12 Physical Component Scale.

#### Monthly SMS Text or 1-Page Questionnaire for 3 Items, Including the Primary Outcome

In addition to the primary outcome (pain intensity), the monthly SMS text or 1-page questionnaire includes 2 potential mediating variables using the following single items for psychological distress and self-efficacy, which are taken and adapted with permission from the validated MSK Health Questionnaire (MSK-HQ) [34]:

“How much distress have you been experiencing because of your pain, on average, over the last 2 weeks?” (Responses were recorded on a scale of 0-10, where 0 is no distress and 10 is extremely distress.)

“How confident have you felt about managing your pain by yourself (eg, medication, changing lifestyle)?” (Responses were recorded on a scale of 0-10, where 0 is not at all confident and 10 is extremely confident.)

### Clinician Behaviors via Linked Medical Records

Clinician decision making and behaviors will be examined through a review of the practice computerized medical records for all patients who give consent for this (at the end of the initial questionnaire). This will allow data to be analyzed from (1) individual patient outcomes, (2) the initial patient-clinician consultation electronic template, and (3) further aspects of their medical record over 6 months following the MSK consultation. Variables of interest from the MSK consultation will include the date of consultation, coded reason for the consultation, MSK pain intensity and location, Start MSK Tool (clinician-completed version) individual items and total score (intervention arm only), and information about the management decisions and other actions taken by the clinician. Other clinical behaviors of interest are described in the *Outcomes* section. The information collected on the patient’s risk subgroup and management options in the intervention practices will be audited and fed back to clinicians at regular intervals, allowing them to see how closely they have followed the matched clinical management options. At the end of the trial, we will also report the fidelity of clinicians in the intervention practices in terms of completing the tool and choosing matched treatment options. The template MSK pain intensity score will also provide the initial score for the primary outcome for participants in both arms of the trial.

Physiotherapists treating patients referred from participating practices will complete their usual clinical records. At the end of the trial, we will collect details about the physiotherapy treatment provided for consenting trial participants to compare between intervention and control.

### Practice-Level Anonymized and Aggregated Data of Musculoskeletal Read Codes

Each participating general practice will provide anonymized medical record data from potentially eligible patients for whom the template was activated through entry of an MSK Read code (estimated n ≥5000). We will compare the following:

1. The characteristics of those patients in which the template is activated with those who respond to the initial questionnaire and provide individual-level patient outcomes. The information examined will not involve any patient-identifiable data and will not be linked to any other data unless prior patient consent has been provided.

2. Aspects of clinical behaviors for 6 months following the index consultation to compare intervention and control practices for key treatment processes for each risk subgroup. For example, this will include requests for the following:

- Prescriptions (eg, categorized into simple analgesics, nonsteroidal anti-inflammatory drugs, neuromodulators, muscle relaxants, corticosteroid injections, and opioids)
- Referrals (eg, categorized into physiotherapy/MSK interface services, specialist services including orthopedics, pain clinics, and rheumatology)
- Imaging (eg, categorized into x-rays/magnetic resonance imaging (MRI) scans, MSK ultrasound scans, and bone density scans)
- Sick certifications or fit notes (eg, categorized into number per patient and mean length in days)
- Repeat MSK general practice visits.

The collection of anonymized and aggregated medical record data is not uncommon within similar general practice research studies that examine potential recruitment bias [35] or for intervention studies examining clinician decision making and behaviors during the consultation (eg, the Primary Care Osteoarthritis Screening Trial [28] and the Study of Work and Pain trial [30,36]).

### Patient and Public Involvement Engagement

This study was discussed and shaped with patient and public involvement engagement (PPIE) through dedicated workshops before the funding submission. The PPIE group agreed with the importance of developing a more robust research base for treatments that can improve the primary care management of MSK pain. Their discussions informed the design and piloting of the text message system and 1-page postal questionnaire used to capture the primary outcome of pain intensity. They also reviewed and improved the patient-facing documentation for the study. Members of the group have expressed an interest in being involved in the analysis of the qualitative data, and it is intended to include them in that process.

Further PPIE meetings were held following the feasibility and pilot trial to identify improvements for the main trial. Their recommendations included the following:

- Updating the invitation pack to provide greater clarity to patients about what is involved in taking part in the trial.
- Simplifying the consent form in the initial patient questionnaire.
- Removing the prize draw system used for participants in the feasibility and pilot trial. This was considered to potentially be confusing for patients and did not appear to lead to a higher response rate to the questionnaires than those in similar research studies.

### Outcomes

#### Primary Outcome

The primary outcome for the trial is the patient-reported clinical outcome of pain intensity, measured monthly over 6 months. Pain intensity is a recommended outcome for trials of MSK pain [37] and had strong face validity among members of the PPIE group. In addition, analysis of our previous MSK cohort data confirmed that this outcome is sensitive to changes in this population.

#### Secondary Outcomes

Secondary clinical outcomes captured at the initial and 6-month questionnaire include body site–specific physical functional measures, using the Roland-Morris Disability Questionnaire for patients with back pain [38], the Neck Disability Index [39,40] for patients with neck pain, the Shoulder Pain and Disability Index [41] for patients with shoulder pain, the Knee Injury and Osteoarthritis Outcome Score-Physical Function Short form [42] for patients with knee pain, and the Short Form 12v2 Physical Component Scale [43] for patients with multisite pain. Other clinical outcomes will include patients’ risk of persistent disabling pain using the Keele Start MSK Tool, and the impact and severity of their MSK pain using the MSK-HQ [34], which includes measures of pain interference with sleep, physical activity level, hobbies/leisure activities, work and daily routine, and quality of life with items for patients’ confidence in managing their pain (pain self-efficacy) and emotional health and understanding of how to deal with their condition. We will also collect fear-avoidance beliefs using the 11-item version of the Tampa Scale of Kinesiophobia [44], and the patient-perceived level of reassurance from their clinician will be captured using the Holt and Pincus [45] reassurance scale, which has 4 subscales: information gathering, relationship building, generic reassurance, and cognitive reassurance. Other outcomes will include health-related quality of life using the EQ-5D-5L to calculate QALYs in the health economic evaluation [46] and single-item questions to capture patient satisfaction with care received, receipt of written education material from their clinician, and overall rating of change in their MSK pain since their primary care consultation [47]. As completion of the initial questionnaire can occur up to 4 weeks following the index MSK GP consultation, this means the first measurement of secondary outcomes is after the commencement of treatment. We chose the timing of the final outcome to be at 6 months as our pilot trial [21] demonstrated a plateau in mean outcomes before this time point. A summary of the patient data collection variables is listed in Table 1.

### Baseline Population Descriptors

To help describe the population recruited, additional baseline descriptors will capture health literacy using the Single Item Literacy Screener [48], episode duration of MSK pain by asking time since last whole month free from this pain, age, sex, employment, and their most recent paid job title (to calculate their socioeconomic status).

### Health Care Resource Use

Questions on additional health care resource use and patient-borne costs including MSK pain–related hospital inpatient stays, outpatient attendance (eg, physiotherapy), other NHS and private practice health care appointments, and over-the-counter medicines and treatment will be included in the 6-month questionnaire. Work performance will be assessed through a single-item work presenteeism question, and time (days) off work will be aligned to occupational information to ascertain the cost of absenteeism.

### Process Evaluation

A process evaluation is planned to explore a range of potential factors that might influence differences between trial arms as well as to better understand how stratified care is used and perceived by patients and clinicians. Following recent Medical Research Council guidance on process evaluations for complex interventions [49], we have designed a mixed methods approach [50]. This will use quantitative analyses focusing on a priori hypothesized intervention targets and qualitative approaches using focus groups and interviews.

A key aim of the process evaluation is to better understand the role of potential intervention targets (mediators) on differences in outcomes between the trial arms [51]. The evidence from our previous stratified care trial (for back pain, the Start Back trial) suggested that the identification and targeting of psychological distress among patients at high risk led to improved outcomes [52]. In addition, a systematic review has recently summarized available evidence and identified pain self-efficacy as another potential mediator [53]. Evidence from the Keele Implementation to Improve Patient Care through Targeted treatment Back study [20], which sought to implement our stratified care approach for patients with low back pain consulting in general practice, suggested that important clinical behavior changes included more systematic identification of patients who are *at risk* of persistent disabling pain who need additional support (leading to more referrals to physiotherapy). After careful consideration by the trial team, a number of potential treatment mediators have been identified a priori, including 3 potential factors at the patient level: (1) reduction in levels of psychological distress measured each month with a single item, (2) increases in pain self-efficacy measured each month with a single item, and (3) the extent of patient-perceived reassurance during the index primary care consultation measured via the initial questionnaire. Changes in these potential patient-level treatment mediators will be examined within a mediation analysis using causal modeling techniques (eg, structural equation modeling) to confirm if they are in the causal pathway explaining any observed between-arm differences in outcome with results also examined at each subgrouping level (low, medium, and high).

In addition, we have identified a number of a priori potential mediators at the level of clinical behavior, measured using the medical record data, including the proportion of patients who receive prescription medications for MSK pain, referrals to other services (eg, physiotherapy and secondary care specialists), referrals for investigations (eg, radiographs, MRI/Computerized Tomography (CT) scans, blood tests), sick certifications (fit notes), and further MSK-related consultations. We will test if there are significant differences in these behaviors between intervention and control practices and whether any of these differences are associated with the results in terms of patients’ pain intensity.

### Sample Size

In an average-sized UK general practice (6000 registered adults), we expect that about 800 potentially eligible patients will consult with the MSK pain sites of interest per year or 400 over 6 months. The feasibility and pilot trial showed that, on average, the template was activated 375 times over 6 months in each practice, and clinicians fully completed it in 41.1% (154/375) of cases (6 times per week), leading to a letter inviting the patient to participate in the data collection. From this, we expect that 40% of patients invited will return their initial questionnaire in the main trial, be eligible, and consent to further data collection (or 62 over 6 months in 1 practice). However, to be more cautious, given the general uncertainty in data and in generalizability of pilot estimates, we have conservatively estimated the average number of participants recruited per practice within 6 months in the main trial to be around 33% of those invited (or n=50 in 6 months or n=9 per month per practice).

The trial is powered at 90% to test the hypothesis of the overall superiority of stratified primary care versus usual care based on an alpha of 5% (two-tailed) to detect a small *effect size* (standardized mean difference) of 0.2 [54] in the primary outcome (pain intensity). An effect size of 0.2 was considered to be appropriate based on information from the feasibility and pilot trial, in which the proportion of responders in the 3 risk subgroups was 32% at low risk, 55% medium risk, and 13% high risk. Our previous trial of stratified care for patients with low back pain (the Start Back trial) found an effect size of 0.3 and 0.4 in the primary outcome (back pain–related physical function) in patients at medium and high risk, respectively. Therefore, we have assumed these standardized differences in this new trial [19]. In addition, the minimal clinically important difference for the numerical rating scale (NRS) pain intensity scores in MSK pain has been reported to be 1 point [55], which equates to an effect size of about 0.4, relative to an expected SD of about 2.5 [54]. We expect that there would be little or no difference between stratified care and usual care for patients in the low-risk subgroup. Hence, by multiplying these effects by the expected proportion within each subgroup, the overall effect size of interest is 0.2 (equating to an absolute mean difference of approximately 0.5 in pain intensity on a 0-10 scale).

The sample size calculation takes into account the clustering of individual participants by practice and likely participant dropout over a 6-month follow-up (inflationary effects on sample size requirement) as well as repeated measurements and adjustment for corresponding baseline pain intensity score (deflationary effects). We have allowed for an Intraclass correlation coefficient of 0.01 based on previous patient-level data from primary care trials [56] as well as expected variation in recruitment per practice using a guideline coefficient of variation of 0.65 [57], and together with an expected loss to follow-up across all time points of approximately 25%, these factors combine to give a sample size inflation factor of ×2.3 (based on an average cluster size of approximately 50 participants per practice in 6 months). The correlation of data within 6 repeated measurements and correlation of follow-up scores with baseline score are typically 0.7 and 0.5, respectively [58], which combine to give a sample size deflation factor of ×0.5. The product of inflation and deflation effects results in a magnification of 1.15 compared with a conventional, individual patient, single follow-up comparison, whereby the sample size requirement would be 525 per trial arm (or 1050 in total). The adjusted sample size target is, therefore, 600 patients per arm (1200 in total) from 24 general practices (12 per arm).

### Statistical Reporting

Data will be reported according to the Consolidated Standards Of Reporting Trials (CONSORT) 2010 statement [59,60], including extensions to cluster randomized trials [61] and pragmatic trials [62].

Final analysis will be carried out after all the data are collected, entered, and cleaned according to Keele CTU SOPs. A flow diagram will show the flow of participants through the trial, including reasons for not taking part and loss to follow-up (split by trial arm). For trial participants, summaries of continuous variables will comprise the number of observations used, mean, median, SD, and interquartile range as appropriate for the distributional form of the data (in total and split by treatment arm). Summaries of categorical variables will comprise the number of observations used and the number and percentage of observations in each category.

Inferential analyses will include reporting of the main (point) estimate for the mean between-arm difference (numerical outcomes) or odds ratio (categorical outcomes) along with 95% CI and *P* values (two-tailed). Odds ratios will also be converted to absolute risk differences (using the usual care prevalence as the base reference in any conversion). Hypothesis tests will use a two-sided 5% significance level. The main analyses will be performed independently by the trial statisticians using the protocol, and the statistical analysis plan agreed with the TSC. For any results discordance(s), if consensus agreement cannot be reached, then a third (independent) statistician will be asked to review and resolve any differences.

### Methods of Analysis

#### Descriptive Statistics: Baseline Characteristics

The baseline demographics and clinical characteristics of general practices and individual participants will be reported. The CONSORT guidelines generally do not recommend statistical significance testing of baseline imbalances between trial arms. However, a more recent publication suggests baseline testing of individual-level characteristics for cluster RCTs to examine the level of selection bias as indicated by potential imbalances in baseline covariates between arms [63].

#### Main Analysis of Primary Outcomes

To avoid any potential bias in the analysis, intention to treat (ITT) will be the primary analysis population (including primary and secondary outcomes) unless otherwise stated in the detailed statistical analysis plan (available from the authors). This is defined as general practice clusters (and affiliated participants) being analyzed as they are randomized regardless of the intervention. Data for individuals who withdraw consent to participate in data collection will be included up to the point of withdrawal. Primary analysis will compare mean differences in pain intensity scores between trial arms over a 6-month follow-up using a hierarchical linear mixed regression model evaluating repeated measures data at 1-, 2-, 3-, 4-, 5-, and 6-month follow-up (level 1) within individuals (level 2) and taking into account clustering of individuals within general practices—the unit of randomization (level 3). The analyses will be adjusted for age, sex, and baseline pain intensity score (recorded from the IT template at the point of consultation) at the individual-patient level and general practice size. This analysis fulfills the ITT principle with analysis as randomized and missing data being accounted for under the missing at random assumption. Although the primary analysis will focus on the *average* intervention effect across 1 to 6 months of follow-up, we will also use treatment by time interaction terms to evaluate between-arm differences in mean responses across each of the individual time points of 1, 2, 3, 4, 5, and 6 months. Model fit will be assessed across different covariance structures (unstructured, independence, exchangeable, autoregressive) to ascertain the best-fit model that will be implemented (ie, the model that gives the lowest Bayesian Information Criterion, Akaike Information Criterion, and highest log-likelihood statistics). The monthly pain intensity scores will be used; however, if, for any individual, the last monthly SMS/brief questionnaire response is missing but they have completed the corresponding pain intensity question in their returned 6-month questionnaire (if completed within 20 days of the date of issue of their monthly SMS/brief questionnaire), then the available pain intensity score response will be used (as the 6-month score) for purposes of the primary outcome evaluation.

#### Analysis of Secondary Outcomes

Analysis of secondary outcomes will be carried out using the ITT approach and using a linear mixed model for numerical outcomes and generalized mixed logistic models for categorical outcomes (adjusted for age, sex, baseline pain, and corresponding baseline score [where applicable] at the individual-patient level and general practice size). For monthly follow-up measures of distress and confidence in managing pain, the analysis will follow that of the primary analysis with initial focus on *average* scores over the 6 months of follow-up and then the time-specific between-arm estimates. The focus of the other secondary measures is on 6-month follow-up data only, with the exception of perceived reassurance, which is captured in the baseline questionnaire.

#### Sensitivity Analysis of the Primary Outcome

A sensitivity analysis will be carried out using a complier average causal effect analysis (CACE) to provide an unbiased estimate of intervention effect for patients treated according to the stratified care protocol, that is, the intervention arm *protocol* is taken as clinical management in line with the recommended matched treatment options for each risk subgroup. CACE will be performed using a 2-step instrumental variable regression modeling approach where the first step relates to model prediction of *compliance* (at level 2 [individual patient level]) using trial arm only as a fixed-effect predictor and practice and participant IDs as random effects, and the second step estimates the between-arm difference in outcome (*average* pain intensity) based on predicted compliance—the endogenous (instrumented) variable (from the first step) and the exogenous (instrumental) variables of trial arm, age, sex and point-of-consultation pain score using a mixed effects model as used in the primary analysis.

#### Subgroup Exploratory Analysis of Primary Outcomes

Subgroup exploratory analysis of the primary outcome (*average* pain intensity) will be carried out by modeling intervention arm interaction terms within the regression models for (1) risk subgroups (low [reference category], medium, and high risk), (2) single MSK pain (reference) site versus multisite pain, and (3) pain site (back [reference], shoulder, knee, and neck). Subgroup analysis was performed regardless of the results of the primary analysis. The mean between-arm difference (and 95% CI and *P* value) will be computed for each subgroup comparison and visually displayed via a forest plot. The main focus will be on the *average* pain intensity rather than on 3-way interactions of intervention-subgroup-time, but the 3-way interaction results will also be examined (and descriptive results produced by subgroup).

#### Exploratory Mediation Analyses

If there is a significant between-arm difference in the primary outcome (overall pain intensity), then we will carry out exploratory mediation analysis by structural equation modeling to examine (1) which potential mediators are *causal* in effect. (2) if psychological mediators (psychological distress or pain self-efficacy in months 1-5) are on the causal pathway for effect, and (3) if patient-perceived reassurance mediates direct/indirect associations of 6-month pain intensity outcomes.

### Evaluation of the Process Outcomes

Process outcomes will be evaluated through comparison of aggregated anonymized data at the level of the participating general practices, by examining, for example, reconsultation rates for MSK pain over 6 months and referral rates to other services between practices in the stratified care versus usual care arms. In intervention practices, we will also investigate the proportions of patients for whom the electronic template is completed, and matched clinical management options are selected overall and by risk subgroup.

A descriptive analysis will be undertaken of physiotherapist data by the intervention arm (eg, waiting times, number of treatment sessions, and clinical grade of treating physiotherapist).

### Examination of Bias

Selection bias will be examined through scrutiny of the comparability of recruitment rates per trial arm and comparability in general practice and participant characteristics. Further, a comparison will be performed examining the characteristics of patients in which the electronic template is activated but who did not take part in the data collection (nonparticipants) with those who did participate in terms of practice distribution; pain intensity scores; location of MSK pain; and (within the intervention arm) the proportion of patients at low, medium, and high risk of persistent disabling pain (from the practice consultation IT template). Both crude descriptive and inferential statistics will be reported.

Differential attrition between trial arms will be examined and reported descriptively: frequencies for responses by trial arm will be recorded in the descriptive tables. We will compare baseline sociodemographic and clinical variables and (for response ≥1) monthly NRS pain intensity scores across level of completion of NRS pain intensity (level of completion is 0 to 6, where 0 is nonresponse, 1 responded once, and 6 responded to all 6 monthly follow-ups) to ascertain whether pattern of missingness is likely to be *missing completely at random*, *missing at random,* or *not missing at random*. If the overall follow-up rate of the primary outcome is over 5% different between trial arms and the pattern of missing data is *missing at random*, then we will undertake a multiple imputation (MI; via chained equations) analysis inclusive of baseline variables that are observed to be statistically associated with follow-up response. Further, if the pattern of missingness is seen to suggest that it is nonignorable, the MI sensitivity analysis will address missing data imputations with an incremented or reduced value corresponding to the overall baseline SD (thereby mimicking the nonignorable pattern).

### Health Economics

The health economics analysis will determine the cost-effectiveness of stratified care in comparison with usual, nonstratified care over 6 months. A cost-consequence analysis will initially be reported, describing all the important results relating to costs and consequences. Subsequently, cost-utility analysis will be undertaken from an NHS/PSS perspective to determine the cost per additional QALY gained. A broader costing perspective will be considered in a secondary analysis, taking into account NHS/PSS costs, private MSK-related health care costs, and productivity costs associated with time off work.

### Costs

Resource use information will be obtained on primary care consultations (eg, general practitioners and practice nurses), secondary care consultations (eg, hospital consultants and physiotherapists), prescriptions, hospital-based procedures (eg, diagnostic tests, injections, and investigations), length of inpatient stay, and surgery. Patients will be asked to distinguish between UK NHS and private provision. Cost data will be collected via a participant questionnaire at 6 months. Unit costs will be obtained from standard sources and health care providers, including the British National Formulary, Unit Costs of Health and Social Care, and NHS Reference costs [64-66]. Given that MSK pain is associated with significant lost productivity, information will also be collected from participants on occupation status, time off work related to their MSK problem, and reduced work performance (presenteeism). This will enable the calculation of productivity costs, allowing analysis from a broader societal cost perspective. The average wage for each respondent will be identified using the UK Standard Occupational Classification coding and annual earnings data for each job type [67].

### Outcomes

The outcome of interest for the economic analysis is QALYs and will be generated from participant responses to the EQ-5D-5L questionnaire at baseline and at the 6-month follow-up. The crosswalk value set will be applied to patient responses to obtain utility scores, in line with current National Institute for Health and Care Excellence recommendations.

### Data Analysis

The cost-utility analysis will be carried out on an ITT basis, with the aim of estimating the difference in costs and QALYs between the stratified care and usual, nonstratified care arms. Missing EQ-5D-5L and cost data will be imputed using MI techniques [68] to ensure that all trial participants are included in the final analysis. For each participant, a QALY score over the 6-month follow-up period will be estimated using the area under the curve approach [69]. Imbalances in baseline utility (EQ-5D-5L) scores between the stratified care and usual nonstratified care arms will be controlled for using a regression approach [70].

The total health care costs over the study period will be calculated by multiplying the resource items used by the respective unit cost and summing over all items. Differences in mean costs and QALYs between the stratified care and usual nonstratified care arms will be estimated. The data for costs are likely to have a skewed distribution; therefore, a nonparametric comparison of means (eg, bootstrapping) will be undertaken to estimate 95% CIs around costs.

Due to the nature of the trial, methods are required to address clustering in both costs and outcomes and to recognize the correlation between individual- and cluster-level costs and outcomes. The methods currently suggested in the health economics literature are multilevel models (MLM) and the 2-stage nonparametric bootstrap, using the Stata 15 [Stata Corp] command TSB) [71]. For the base case scenario, MLM will be used to estimate differential costs, differential QALYs, and incremental net benefits. The analysis will also allow us to control for covariates. The robustness of the results will be explored using sensitivity analysis. This will explore uncertainties in the trial data itself as well as the methods employed to analyze the data. A cost-effectiveness acceptability curve will be constructed to assess the probability that stratified care is effective at different willingness-to-pay thresholds. To estimate productivity costs, self-reported days off work will be multiplied by the average wage rate. The analysis will use the human capital approach.

Planned sensitivity analysis will include (1) a complete case analysis as an alternative to using an imputed dataset; (2) a broader societal perspective; and (3) additional exploratory analyses that will consider the cost-effectiveness of stratified care versus usual nonstratified care for patients in the low-, medium-, and high-risk subgroups separately. All analyses will be performed using StataCorp 15 software.

### Linked Qualitative Study

#### Theoretical Framework

Two theoretical frameworks will underpin the evaluation. First, the COM-B model [72] offers a way of understanding behavior in the context of complex interventions around 3 key determinants: *capability* (the psychological or physical ability to enact the behavior), *opportunity* (the physical and social environment that enables the behavior), and *motivation* (the reflective and automatic mechanisms that activate or inhibit behavior). Second, normalization process theory provides a framework for understanding how/why some new health care interventions are accepted and taken up, whereas others are less successful [73]. Both frameworks emphasize the broader sociopolitical contexts in which health behaviors and practices are situated and the importance of taking these contexts into account in understanding the adoption of new interventions [73,74].

#### Aim

This study aims to understand the ways in which stratified care is perceived and operationalized, from the perspectives of health care professionals and patients, taking into account individual, local, and national contexts.

#### Objectives

The specific objectives of our linked qualitative research will be as follows:

- Identify the acceptability and impact on the consultation of using the clinician-completed version of the Keele Start MSK Tool and the extent to which the matched treatment options are viewed as being in line with clinical judgments on best practice.
- Understand the impact of stratified care on (1) individual clinicians; (2) general practice and physiotherapy services; (3) interprofessional and professional-patient communication; and (4) patients at low, medium, and high risk.
- Document any variation in experiences or views across different practices and services in the trial.

#### Methods for Linked Qualitative Study

An iterative, mixed methods approach will be adopted [50,75], with the quantitative data informing the qualitative data collection and analysis from both informing the overall findings and conclusions. Data will be drawn from clinicians and patients.

#### Clinicians

GPs and physiotherapists involved in delivering stratified care will be invited to participate in up to 3 separate focus groups held at approximately 4 GP practices. Where clinicians are unable to attend focus groups, arrangements will be made for individual interviews. Initial focus groups/interviews will explore clinicians’ views and experiences of delivering stratified care during the course of the trial. Follow-up focus groups/interviews will be conducted at a later stage once trial results are available to explore views on the trial results and, depending on these results, discuss potential implications for practice, policy, and service provision beyond the trial.

#### Patients

One-to-one semistructured interviews will be conducted to explore individual patient experiences. Patients at low risk will be interviewed approximately 2 months after their index primary care consultation, whereas patients at medium and high risk will be interviewed at approximately 4 months. This timescale will allow participants to reflect on their experiences of clinical management (including time to access any treatments), communication with the clinicians involved in their care, and their health care resource use over time.

### Sampling

Clinicians and patients will be sampled from the stratified care arm of the trial. GPs directly involved in the trial will be identified based on the diversity of practice characteristics, including size and geographic location. A sample of physiotherapists in linked participating services will also be invited to participate. Patients will be purposively sampled from baseline questionnaire responses to capture diverse characteristics, such as pain scores and health-related quality of life, risk subgroup, comorbidity, age, sex, and socioeconomic status.

### Sample Size of the Qualitative Study

Data collection will continue until saturation is reached, defined as *informational redundancy*, ‒ the point at which additional data no longer offer new insights [76]. We estimate that around 20 to 30 clinicians and approximately 20 to 30 patients will be required.

### Recruitment to a Qualitative Study

*Clinicians* will be informed that as part of their participation in the trial, they may be approached to participate in focus groups or interviews. Additional information explaining confidentiality, anonymity, data storage, and archiving will be distributed ahead of each focus group/interview and individual written consent obtained before the start of the discussion.

*Patients* will be informed that, as part of their participation in the study, they consented to further research contact. An invitation letter and detailed interview information leaflet will be mailed to the patient, and after 2 to 3 days, a researcher will telephone the patient to check if they are willing to participate and, if so, make arrangements for the interview. Interviews may be face-to-face or by telephone, based on participant preference, and will be arranged at a time/location convenient for the participant. Once an interview has been arranged, a confirmation letter will be sent. Written consent will be obtained at the start of the interview or audio-recorded if the interview is via telephone and checked again at the end. Interviews are estimated to last approximately 1 hour.

### Trial Management, Study Administration, and Data Storage

The trial manager assisted by the study coordinator will oversee the day-to-day running of the trial. General practice staff assisted where necessary by the CRN will download details of patients who have a completed template (name, address, MSK pain site, date of MSK consultation, and EMIS patient identification number) on a weekly basis from each practice. Practice staff will arrange transfer of patient details to the dedicated research administrator in Keele CTU using nhs.net-to-nhs.net transfer for mailing of the study invite packs to potential participants. A unique study number will be applied to each potential participant. On return of a completed initial questionnaire, details will be entered into the research database to ensure that no unnecessary reminders are sent. Details of informed consent will be stored in the research database, including participants’ names and contact details. In this database, participants will be primarily identified by study number. Data will be entered into the research database by trained members of the administrative team who will be blinded to general practice allocation. Access to the database will be restricted to those members of the team that require access. The coding schedule for the questionnaires will be used to inform the database design and to facilitate data entry. Details of data entry accuracy will be kept by the research data management lead and trial statisticians and reported.

Any requests for access to the anonymized data will follow our data-sharing procedure. Requests for anonymized data will be reviewed by our Data Custodian and Academic Proposals Committee. The full statement on data sharing is publicly available [32]. All information will be held securely and in strict confidence. Each person in this study will be given a study number so that data from the study will not have any identifiable information, such as names and addresses, and cannot be traced. On this basis, these *anonymized* data will be kept electronically and may be used in other research studies.

### Clinical Governance Issues

To ensure responsibility and accountability for the overall quality of care received by participants during the study period, clinical governance issues pertaining to all aspects of routine management will be brought to the attention of the TSC and, where applicable, to individual participating practices or NHS services. One potential issue is that GPs in the intervention arm may feel that the recommended matched clinical management options are not appropriate for an individual patient, in which case they will need to choose a treatment that is not among the recommended options. The clinician training sessions will make it clear that despite being part of a clinical trial, clinicians retain the responsibility to provide appropriate care to their patients. Clinicians will be encouraged to report to the research team where there are consistent difficulties with the stratified care intervention.

### Statement of Indemnity and Trial Sponsors

Keele University has in place clinical trial indemnity, which provides coverage to the university for harm that comes about through the university’s or its staff’s negligence in relation to the design or management of the trial and may alternatively, at the University’s discretion, provide cover for nonnegligent harm to participants. The NHS has a duty of care to patients treated, whether or not the patient is taking part in a clinical trial, and the NHS organization (general practices and other services involved) remains liable for clinical negligence and other negligent harm to patients under this duty of care. The sponsor (Keele University) is responsible for trial initiation management and financing of the trial as defined by Directive 2001/20/EC. 

### Oversight/Trial Monitoring

The Trial Management Group (TMG) comprises the chief investigator, associate investigator, Keele CTU staff, and other key trial team members. They are responsible for the clinical setup, ongoing management, promotion of the trial, and analysis and interpretation of results. Specifically, the TMG is responsible for (1) protocol completion; (2) study document development; (3) obtaining health research authority approval; (4) completing cost estimates and project initiation; (5) facilitating the TSC and DMC; (6) reporting of serious adverse events (SAEs); (7) monitoring of recruitment, intervention, and follow-up procedures; (8) data collection; and (9) database development. The group will meet on a regular basis, typically monthly, throughout the trial. The trial does not incorporate any a priori stopping rules, and hence, no planned interim analysis of the outcome measures collected in the trial will be carried out.

### Financial Arrangements

Clinicians participating in the focus groups/interviews will receive a reimbursement of their time using standard professional rates. Patients participating in an interview will be given a GBP £10 (US $12.2) Love to Shop gift token by way of thanking them for their participation and will only receive remuneration for travel if they participate in an interview at a site other than their home.

### Serious Breaches of the Protocol and Good Clinical Practice

Keele CTU has systems in place to ensure that serious breaches of GCP are picked up and reported. A *serious breach* is a breach that is likely to effect to a significant degree: the safety or physical or mental integrity of the participants of the trial or the scientific value of the trial. All protocol deviations or breaches of the GCP will be recorded and reported to the sponsor according to the relevant SOP.

### Serious Adverse Events

The NHS Research Committee approval reference is 16/EM/0257. Patient participants gave written consent to participate. SAEs include death, hospitalization, significant disability or incapacity, any life-threatening circumstance, or any other medically significant occurrence that is believed to be related to the trial or interventions. All participating practice staff and physiotherapists will be asked to report as soon as possible to the chief investigator any SAEs among patient participants, that are likely to be related to the trial. We have discussed this issue with the independent TSC and agreed that the potential harms of the study are considered to be minimal and the stratified care information and matched treatment options are considered not only to be evidence-based but also have strong clinical community endorsement and credibility. Any SAEs will be brought to the immediate attention of the trial team. The chief investigator will then assess whether the event was related to or resulted from any of the trial procedures or interventions, according to the process laid out in Keele CTU’s SOPs. Any unexpected SAE considered to be related to the trial procedures will be reported to the main research ethics committee by the chief investigator within 15 days of becoming aware of the event. In addition, all such events will be reported to the trial sponsor, TSC, and DMC.

### Confidentiality and Anonymity

All information collected during the course of the trial will be kept strictly confidential. All identifying information will be anonymized before being used for analysis. Information will be held securely on paper and managed electronically by Keele University through Keele CTU. Keele CTU complies with all aspects of the 1998 Data Protection Act. The trial data will be held on a database hosted on a secure server by Keele CTU. All research staff involved in this study adhere to robust data security procedures and have explicit duties of confidentiality. These practices are written into their employment contracts and are equivalent to the duties placed on NHS staff. If a participant withdraws consent from further collection of data, their data collected to date will remain on file and will be included in the final study analysis unless requested otherwise.

## Results

The trial was funded as part of a 6-year research program in June 2014, the pilot trial was undertaken from October 2016 to May 2017 and the main trial was approved by research ethics committee in February 2018. Data collection for the main trial commenced in May 2018, and ended in July 2019, after a recruitment period of 14 months in 24 GP practices, which successfully recruited 1203 patient participants. All 6-month follow-up and interview data collection was completed in February 2020. Data analysis is currently in progress with expected results to be published in summer 2020. There have been no important changes to methods since trial commencement.

## Discussion

This study protocol describes the details of the Start MSK trial, which aims to investigate the clinical and cost effectiveness of stratified primary care for patients with the 5 most common MSK pain presentations compared with usual nonstratified care. The intervention was designed to improve patient outcomes including pain intensity, physical function, and quality of life as well as clinician decision making to reduce treatment variability and improve adherence to best practice. This trial is the first attempt, as far as we know, at testing a prognostic stratified care approach for primary care patients with MSK pain. The results of this trial should be available in the summer of 2020.

## Appendix

Multimedia Appendix 1 CONSORT-eHEALTH checklist (V 1.6.1).

## Abbreviations

CACE: causal effect analysis
CRN: Clinical Research Network
CTU: Clinical Trials Unit
DALYs: disability-adjusted life years
DMC: Data Monitoring Committee
EMIS: Egton Medical Information Systems
EMR: electronic medical record
EQ-5D-5L: EuroQol-5D-5L
GP: general practitioner
ITT: intention to treat
MI: multiple imputation
MLM: multilevel models
MRI: magnetic resonance imaging
MSK: musculoskeletal
MSK-HQ: MSK Health Questionnaire
NHS: National Health Service
NIHR: National Institute for Health Research
NRS: numerical rating scale
PIL: patient information leaflet
PPIE: public involvement engagement
PSS: Personal Social Services
QALYs: quality-adjusted life years
RCT: randomized controlled trial
SAEs: serious adverse events
SOPs: standard operating procedures
TMG: Trial Management Group
TSC: Trial Steering Committee
